# Pretibial myxedema in Grave's disease: A case report and treatment review of the literature

**DOI:** 10.1002/ccr3.8478

**Published:** 2024-02-22

**Authors:** Van Bang Nguyen, Van Vy Hau Nguyen, Chi Van Le, Pham Nguyen Tuyen Linh, Xuan Nguyen Thi, Thanh Trang Vo

**Affiliations:** ^1^ Center of Endocrinology and Diabetes Family Hospital Da Nang Vietnam; ^2^ Internal Medicine Department, Hue University of Medicine and Pharmacy Hue University Hue City Vietnam

**Keywords:** case report, hyperthyroidism, pretibial myxedema, thyroid‐stimulating hormone receptor antibodies, topical corticosteroids

## Abstract

**Key Clinical Message:**

Pretibial myxedema is a rare skin lesion in Grave's disease, which required topical glucocorticoid administration in long‐term treatment. The patient's lesion has shrunk and become flatter than before treatment.

**Abstract:**

We present a case of biopsy‐verified pretibial myxedema in a 70‐year‐old male patient with diagnosed hyperthyroidism and no prior history of Graves' disease. Topical corticosteroid and antithyroid drug administration led to successful resolution of the skin lesions. This case emphasizes the importance of considering pretibial myxedema even in atypical presentations of Graves' disease and underscores the value of prompt treatment.

## Graphical abstract

Comparison between before and after treatment of pretibial myxedema.

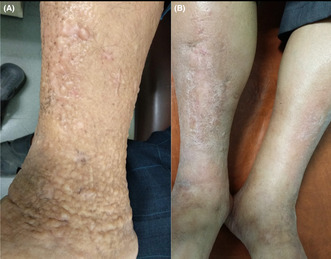

## 1 INTRODUCTION

Pretibial myxedema (PTM) is an uncommon autoimmune skin manifestation of Graves' disease (GD). It results from the accumulation of hyaluronic acid (HA) in the dermis and subcutaneous tissue due to the stimulation of cytokines. HA, the main glycosaminoglycan in the skin, has a high water‐binding capacity (hygroscopicity) and can increase its volume up to 1000 times its original state. This explains the characteristic clinical appearance of pretibial myxedema.^1^ Thyroid dermopathy, the broader term for pretibial myxedema and other localized skin manifestations of GD, typically appears on the front and sides of the lower legs. However, it can extend to other areas such as the knees, elbows, shoulders, neck, and upper back.^2^ PTM is a relatively rare and late clinical manifestation of GD, with a prevalence of 0.5%–4.3%. This prevalence can increase to 15% in GD patients with associated ophthalmopathy.^3^ A key factor in the pathogenesis of dermopathy is the presence of Thyroid‐stimulating hormone (TSH) receptors in the skin's fibroblast. TSH receptor autoantibodies (TRAb) present high serum concentrations in all patients with localized myxedema.^4^ The presence of typical pretibial lesions, Graves' ophthalmopathy, and a history of thyrotoxicosis usually make the diagnosis of localized myxedema clear‐cut. However, in atypical cases with a euthyroid state, a biopsy of the lesions can be helpful for diagnosis.^3^

Treatment for thyroid dermopathy is not always necessary. In many cases, the lesions improve or even resolve spontaneously after thyroid function is restored to normal levels. Regarding the treatment of cosmetic issues, functional problems, or localized discomfort, local corticosteroid therapy should be used as the first step. The less severe and extensive the lesions are, the more effective the treatment is.^5^ It is recommended to use adjunctive treatments such as radiotherapy, surgery, gamma globulin, intralesional steroids, and pentoxifylline. Local compressive therapy may provide additional benefits in cases of severe edema and elephantiasis.^6^ We report a case of a 70‐year‐old patient with Grave's disease.

## 2 CASE REPORT

A 70‐year‐old male patient presented to the clinic with a long‐standing erythematous, non‐pitting edema on the pretibial regions. He had been experiencing these symptoms for about a year without a diagnosis. His 10‐year medical history included hypertension, carotid artery atherosclerosis, and hyperlipidemia, treated with medications such as telmisartan combined with hydrochlorothiazide (40/12.5 mg daily), rosuvastatin (10 mg daily), and clopidogrel (75 mg daily). He had no history of smoking or alcohol consumption. The family history was unremarkable.

The clinical examination recorded blood pressure of 120/80 mm Hg, heart rate of 80 beats/min with regular rhythm. He looked well, no symptoms of sweating and fatigue. His thyroid gland was not palpable. Ophthalmopathy symptoms such as bulging, dryness, inflammation, or impaired vision, were not present. The patient presented with plaques resembling the shape of his shoes on both the medial and lateral sides of his bilateral pretibial areas. These plaques manifested as firm, non‐pitting edema with a peau d'orange appearance. These are the main clinical features of pretibial myxedema, a manifestation of Graves' disease. While the patient displayed hyperthyroidism, other hallmark symptoms of GD, including weight loss, hyperhidrosis, and tachycardia, were absent. With TSH levels less than 0.005 IU/mL (reference range: 0.27–4.2 IU/mL), free thyroxine (T4) of 1.32 ng/dL (reference range: 0.93–2.33 ng/dL), a thyroid function test confirmed subclinical hyperthyroidism. Besides, he had thyroperoxidase positivity of more than 600 IU/mL of antibodies (reference range: 0–34 IU/mL), TRAb positivity with more than 40 IU/L (reference range: 0–1.22 IU/L), and a negative antinuclear antibody test. Plasma glucose, lipid profile, serum creatinine, urine test, liver function tests, and biochemical tests were all within normal ranges.

**FIGURE 1:**
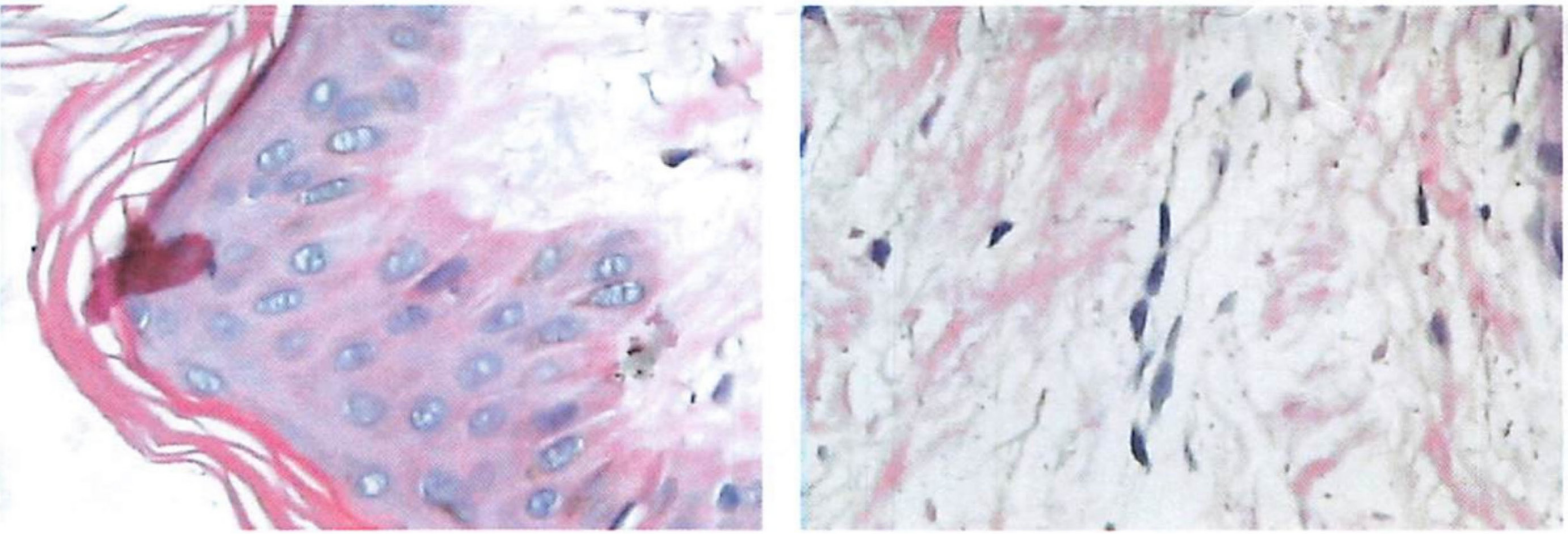
Immunohistochemical staining results.

A comprehensive thyroid ultrasound examination was performed, demonstrating diffusely heterogeneous echogenicity without any detectable nodules. Both lobes measured 2.5 cm in thickness and 5.2 cm in length, and the isthmus depth was 1 cm. Doppler color flow showed increased vascularization within the thyroid gland. Histopathological analysis of the 5‐mm punch right lesion skin biopsy samples, stained with periodic acid‐Schiff (PAS) and Alcian blue, revealed mild hyperkeratosis characterized by abundant mucin deposition, and thin collagen fibers in the dermis (Figure 1).

**FIGURE 2:**
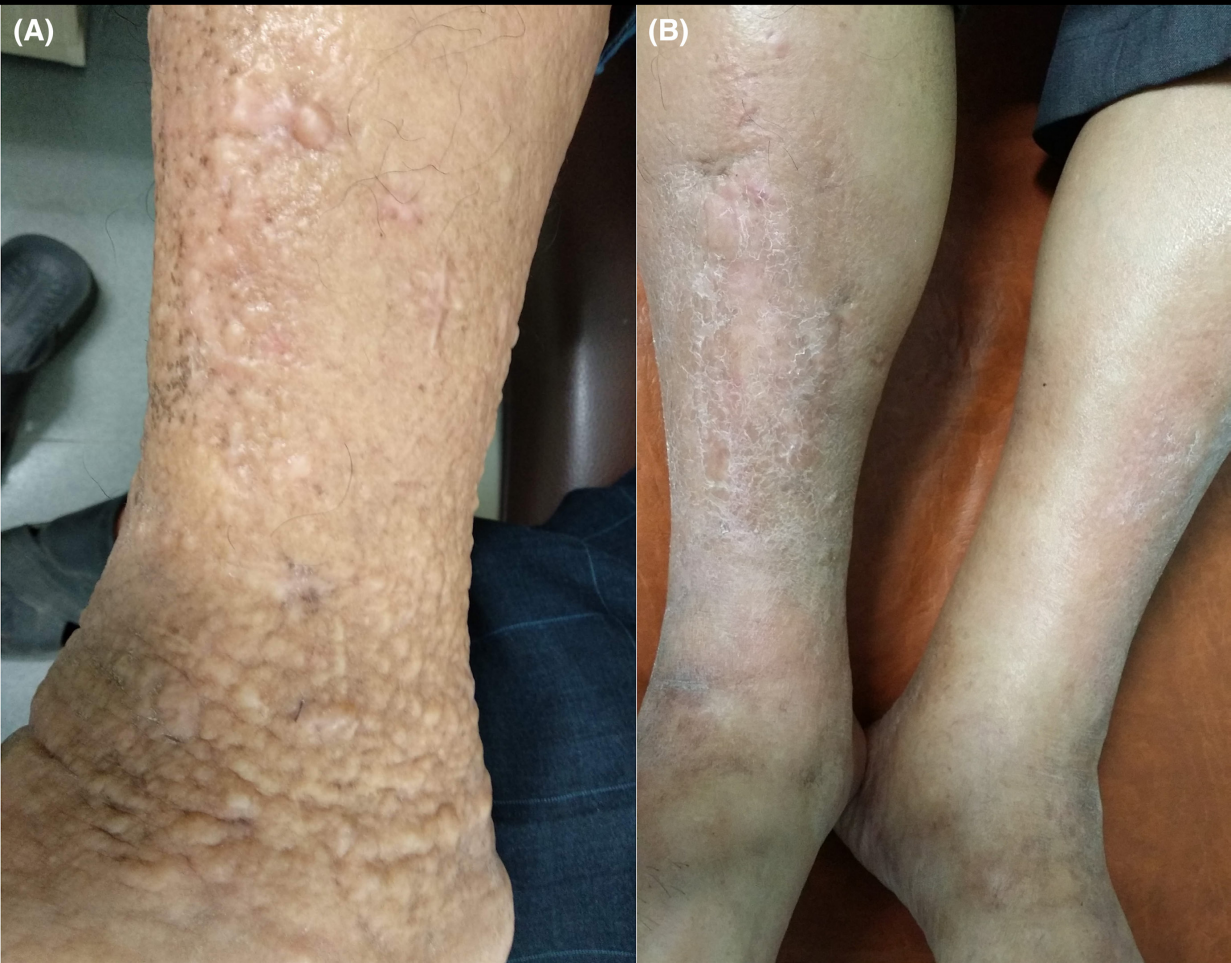
Skin lesions before (A) and after (B) successfully applying corticosteroids.

Prior to treatment, the patient exhibited mild bilateral, non‐pitting periorbital edema in the absence of exophthalmos (Figure 2A). Pretibial myxedema (PTM) was initially managed with a combination of antithyroid medications, a beta‐blocker, and topical betamethasone. A topical corticosteroid was applied directly to the lesions each morning and maintained with frequent application for 3 years. Euthyroidism was achieved, and the pretibial lesions on both sides demonstrated significant improvement and resolution. The patient's lesion has shrunk and become flatter than before treatment (Figure 2B).

## 3 DISCUSSION

PTM is a significant GD‐specific skin manifestation. Clinically, it appears as skin‐colored to yellow‐brown to erythematous nodules and plaques that are bilateral, asymmetrical, firm, non‐pitting, and painless. Pretibial skin involvement is the most prevalent. The lesions typically appear after the onset of clinical hyperthyroidism; however, PTM can happen at any stage of GD, even years after the hyperthyroidism has been successfully treated.^7^

Lymphocytic infiltration, which is best visible in early lesions, gives rise to cytokines. Mucinous edema and the fragmentation of dermal collagen fibers with subsequent extension into deeper tissue are two of the resulting distinctive pathologic transformations.^8^ The antibody responsible for the commencement of cellular and humoral autoimmunity is TSH receptor (TSHR), which is found in fibroblasts. An inflammatory response is triggered by TRAb and/or antigen‐specific T cells, which increases HA production.^2^ TNF‐alpha and gamma interferon are cytokines that cause fibroblasts to release HA. These cytokines could be released by T cells of the Th1 subtype that have been stimulated by the TSHR antigen.^9^ Light microscopy reveals that there are many HAs in the biopsy specimens, a small number of lymphocytes, and a moderate increase in mast cells. Typically, extensive lymph cell infiltration is not observed. Hematoxylin and eosin staining reveals collagen fragmentation. The mucinous substance between collagen fibers can be seen using periodic acid‐Schiff stains and Alcian blue. In more severe cases, papillomatosis, acanthosis, and hyperkeratosis may be present.^10^

Because the lesions are typically not symptomatic or particularly unsightly and can be covered by clothing, the majority of patients do not require therapy. Topical corticosteroids are more likely to be used than systemic treatment, given the relatively benign nature of this condition. Topical corticosteroids are offered in a range of potencies, from low‐potency steroids including fluocinolone acetonide.^11^ to high‐potency steroids, such as clobetasole propionate.^12^ With the utilization of hydrocolloid^12^ or occlusive dressings made of plastic wrap, topical corticosteroids' absorption has been further improved. The length of these treatments varies. Occlusion is typically used for at least a dozen hours every day. A 4‐ to 6‐week trial may be reasonable, but it must be carefully monitored for any signs of topical steroid side effects, such as atrophy, telangiectasis, and ecchymoses. Compression has also been effective, particularly when lymphatic involvement is thought to be present.^13^ Compression is best achieved with athletic wraps or stockings that maintain a 20–40 mm Hg pressure. In order to reduce the degree of disfigurement, improve function, prevent tissue breakdown, and treat compressive complications, patients may need to receive treatment for many months. Success with intravenous glucocorticoids followed by oral glucocorticoids has been documented in case reports. The high rate of dermopathy recurrence makes surgical excision generally not advised, despite the success of some case studies. Octreotide, intravenous immunoglobulin, pentoxifylline, and plasmapheresis are some of the treatments that have shown varying degrees of success in small studies.^6^

More than half of patients with mild dermopathy did not receive any treatment experienced full remission in less than 17 years, reported in the largest series of 216 cases, which only treated severe cases.^14^ The outcome of severe cases treated with topical corticosteroids and other therapies was no better than that of milder cases who did not receive any specific therapy. After 25 years of follow‐up, 58% of severe cases treated with local therapy and 70% of milder untreated cases experienced a partial or full remission. Long‐term remission seems to be more a function of the initial disease severity than a result of treatment. Therefore, it is challenging to assess the long‐term effectiveness of these treatments—topical corticosteroids and compressive dressings—from the current studies because they have not been directly compared with the non‐therapy group of patients with severe dermopathy yet.^3^

In our case study, the patient's pretibial lesions were treated with a combination of potent topical corticosteroid and antithyroid medications. Following 3 years of treatment, the lesions in both pretibial regions demonstrated significant improvement and resolution, and euthyroidism was achieved.

## 4 CONCLUSION

In summary, we present a rare instance of pretibial myxedema manifesting in a patient with hyperthyroidism from Grave's disease. The patient is diagnosed based on typical clinical and subclinical examinations with hyperthyroid symptoms, positive TRAb, low serum TSH, and normal serum free thyroxine levels. Additionally, specific histopathology may be confirmed as a diagnostic aid for pretibial lesions, but health facilities may lack access to this technique. A combination of antithyroid drugs and topical steroid mitigates and improves considerably pretibial myxedema.

## AUTHOR CONTRIBUTIONS

**Van Bang Nguyen:** Conceptualization; data curation; funding acquisition; investigation; methodology; resources; supervision; writing – original draft. **Van Vy Hau Nguyen:** Conceptualization; data curation; formal analysis; investigation. **Chi Van Le:** Conceptualization; supervision; writing – review and editing. **Linh Pham Nguyen Tuyen:** Formal analysis; investigation. **Xuan Nguyen Thi:** Conceptualization; data curation; resources. **Thanh Trang Vo:** Conceptualization; data curation; funding acquisition; methodology; project administration; resources; supervision; validation; visualization; writing – original draft.

## FUNDING INFORMATION

Not applicable.

## CONFLICT OF INTEREST STATEMENT

The conflict of interest relevant to this article was not reported.

## CONSENT

Written informed consent was obtained from the patient to publish this report in accordance with the journal's patient consent policy.

## Data Availability

Availability of data and materials supporting our findings will be shared upon request.
